# Actual versus ideal body weight dosing of sugammadex in morbidly obese patients offers faster reversal of rocuronium- or vecuronium-induced deep or moderate neuromuscular block: a randomized clinical trial

**DOI:** 10.1186/s12871-021-01278-w

**Published:** 2021-02-27

**Authors:** Jay C. Horrow, Wen Li, Manfred Blobner, John Lombard, Marcel Speek, Matthew DeAngelis, W. Joseph Herring

**Affiliations:** 1 Department of Clinical Research, Merck & Co., Inc., 2000 Galloping Hill Rd, UG-4C-13, Kenilworth, NJ 07033 USA; 2 Present address: Anesthesiology & Critical Care Medicine, University of Pennsylvania, Philadelphia, PA USA; 3 Department of Anaesthesiology and Intensive Care Medicine, School of Medicine, Technical University of Munich, Munich, Germany; 4 Department of Anaesthesiology and Intensive Care Medicine, Faculty of Medicine, University of Ulm, Munich, Germany

**Keywords:** Recurarization, Bradycardia, Neostigmine, Multicenter trial

## Abstract

**Background:**

This randomized, double-blind trial evaluated sugammadex-mediated recovery time from rocuronium- or vecuronium-induced moderate (M-) or deep (D-) neuromuscular block in morbidly obese adults dosed by actual (ABW) or ideal body weight (IBW).

**Methods:**

Adults with BMI ≥40 kg/m^2^ were randomized to 1 of 5 groups: M-neuromuscular block, sugammadex 2 mg/kg ABW; M-neuromuscular block, sugammadex 2 mg/kg IBW; M-neuromuscular block, neostigmine 5 mg, and glycopyrrolate 1 mg; D-neuromuscular block, sugammadex 4 mg/kg ABW; or D-neuromuscular block, sugammadex 4 mg/kg IBW. Supramaximal train of four (TOF) stimulation of the ulnar nerve (TOF-watch SX®) monitored recovery. Primary endpoint was time to TOF ratio ≥ 0.9 for ABW and IBW groups pooled across neuromuscular blocking agent (NMBA)/blocking depth, analyzed by log-rank test stratified for agent and depth. Prespecified safety outcomes included treatment-emergent bradycardia, tachycardia, and other arrhythmias, and adjudicated hypersensitivity and anaphylaxis.

**Results:**

Of 207 patients randomized, 188 received treatment (28% male, BMI 47 ± 5.1 kg/m^2^, age 48 ± 13 years). Recovery was 1.5 min faster with ABW vs IBW dosing. The sugammadex 2 mg/kg groups recovered 9-fold faster [time 0.11-fold, 95% CI 0.08 to 0.14] than the neostigmine group. ABW (5.3%) and IBW (2.7%) groups had similar incidences of recovery time > 10 min (95% CI of difference: − 4.8 to 11.0%); 84% for neostigmine group. Re-curarization occurred in one patient each in the 2 mg/kg IBW and neostigmine groups. Prespecified safety outcomes occurred with similar incidences.

**Conclusions:**

ABW-based sugammadex dosing yields faster reversal without re-curarization, supporting ABW-based sugammadex dosing in the morbidly obese, irrespective of the depth of neuromuscular block or NMBA used.

**Trial registration:**

Registered on November 17, 2017, at ClinicalTrials.gov under number NCT03346070.

**Supplementary Information:**

The online version contains supplementary material available at 10.1186/s12871-021-01278-w.

## Background

Sugammadex (Bridion®, Merck & Co., Inc., Kenilworth, NJ, USA), a modified cyclodextrin, reverses neuromuscular blockade from the neuromuscular blocking agents (NMBA), rocuronium and vecuronium. Unlike anticholinesterases, which flood nicotinic and muscarinic sites with acetylcholine, sugammadex encapsulates unbound rocuronium and vecuronium providing rapid, predictable reversal, and avoiding anticholinesterase side effects and antimuscarinic drug use.

Morbid obesity (body mass index [BMI] > 40 m^2^/kg) alters anatomy and physiology. Excess neck and pharyngeal adipose tissue make difficult the maintenance of airway patency; increased chest wall mass reduces functional residual capacity, with potential hypoxemia with spontaneous ventilation during anesthesia. Increased risk of reflux and impaired airway protective reflexes increase aspiration risk; and obstructive sleep apnea has post-operative respiratory complications. In morbidly obese individuals, increased lean body weight accounts for 20–40% of the excess actual body weight (ABW) [1, 2], leading to increased cardiac output [3] and drug clearance [4]. Alterations in regional blood flow in drug movement among body compartments impact the pharmacokinetics and pharmacodynamics of anesthetics, including volume of distribution and context-sensitive half-times.

Product labels for sugammadex stipulate dosing by ABW without adjusting for body habitus. The clinical development program for sugammadex dosed by ABW, mirroring ABW-based NMBA dosing, provided a consistent molar ratio of sugammadex: NMBA to limit residual block and re-curarization. For many drugs, appropriate weight corrections in morbid obesity remain unexplored. Ideal body weight (IBW) is that associated with maximum life-expectancy for a given height. For morbidly obese patients, IBW is substantially less than ABW, so IBW-based dosing can under-dose [5]. For reversal agents, under-dosing can cause prolonged recovery, residual neuromuscular block, or re-curarization. For other medications, ABW-based dosing may be excessive and engender side effects. The pooled clinical trials supporting sugammadex registration included 157 obese patients (BMI ≥30 m^2^/kg); no meaningful differences in sugammadex efficacy or safety arose, supporting no dosage adjustment for obesity. However, data in the morbidly obese are limited.

Conducted by Merck Sharp & Dohme Corp. (a subsidiary of Merck & Co., Inc., Kenilworth, NJ, USA) as a post-marketing requirement of the United States Food & Drug Administration (FDA), this study compared the efficacy and safety of ABW-based to IBW-based sugammadex dosing in the morbidly obese after moderate or deep neuromuscular block with either rocuronium or vecuronium. The FDA specified many design elements, including the choice of IBW, a control arm, and including both neuromuscular blocking agents. The primary efficacy measure was time to recovery of train of four (TOF) ratio ≥ 0.9 [6–8]. Safety evaluation included incidences of treatment-emergent bradycardia, tachycardia, other arrhythmias, hypersensitivity, and anaphylaxis.

## Methods

The study was conducted by Merck Sharp & Dohme Corp., a subsidiary of Merck & Co., Inc., Kenilworth, NJ, USA. Ethics committees at each site approved this randomized, active comparator-controlled, parallel-group, double-blind study (Protocol 146; Clinicaltrials.gov: NCT03346070), conducted at 25 sites in 5 countries from January, 2018 to January, 2019. All patients provided written, informed consent. Physician investigators at all sites in the United States were Board Certified Anesthesiologists by the American Board of Anesthesiology or certified to practice anesthesiology in the United States. Participating investigators within the European Union were licensed physicians with specialties in anesthesiology in their respective countries, requiring comprehensive training meeting and/or exceeding requirements in the United States. All participating investigators met Health Authority qualifications to serve as investigators in clinical trials. We performed this randomized study following the recommendations of Consolidated Standards of Reporting Trials (CONSORT) guidelines.

Patients included men and women 18 years or older with BMI ≥40 m^2^/kg and American Society of Anesthesia Physical Status class 3 with planned surgical procedures involving neuromuscular block with either rocuronium or vecuronium. Exclusion criteria were: ABW < 100 kg; pacemaker or implantable cardioverter-defibrillator precluding assessment of arrhythmias; plan not to reverse neuromuscular block at procedure end; neuromuscular disorder affecting neuromuscular block or assessments; severe renal insufficiency (defined as calculated CrCl < 30 mL/min by Cockroft-Gault); history or family history of malignant hyperthermia; known or suspected allergy to peri-operative medications; toremifene application within 24 h (before or after) study drug administration.

The investigator specified the intended NMBA, vecuronium or rocuronium, at enrollment; an automated system stratified randomization by NMBA and capped rocuronium enrollment at 70%. The protocol (Additional file 1) did not specify anesthetic agents for induction or maintenance. Treatment assignment determined the depth of neuromuscular block and study medication for its reversal, randomized equally among 5 maintenance/reversal combinations, stratified by choice of NMBA: (1) moderate neuromuscular block maintenance and reversal with sugammadex 2 mg/kg dosed by ABW; (2) moderate neuromuscular block maintenance and reversal with sugammadex 2 mg/kg dosed by IBW; (3) moderate neuromuscular block maintenance and reversal with neostigmine 5 mg + glycopyrrolate 1 mg; (4) deep neuromuscular block maintenance and reversal with sugammadex 4 mg/kg dosed by ABW; (5) deep neuromuscular block maintenance and reversal with sugammadex 4 mg/kg dosed by IBW. IBW was calculated according to Kammerer et al. [9]. A follow-up contact 14 days after the procedure collected adverse events and events of clinical interest.

For reversal of moderate neuromuscular block, neostigmine was selected as the active comparator because it is the most frequently used acetylcholinesterase inhibitor indicated for reversal of moderate block. As per current prescribing information, neostigmine was co-administered intravenously with glycopyrrolate to counteract the anticipated muscarinic effects of neostigmine, most notably bradycardia. Neostigmine was administered at the dose of 50 mg/kg or up to a total of 5 mg whichever is less [10]. Glycopyrrolate was administered at a dose that proportionally counteracts the muscarinic effects at the ratio of 1:5 at a dose of 10 μg/kg, and thus also capped at 1 mg for a 100 kg patient. The ratio neostigmine:glycopyrrolate is consistent with previous studies in the sugammadex development program and most literature [11–13]**.**

The operating room staff, unblinded to target depth of block in order to achieve it, were blinded to dose assignment. At each site, designated safety assessors, blinded and firewalled to both depth of block and dosing, did not observe preparation of trial medications or enter the operating room during anesthesia. Induction and maintenance of anesthesia proceeded per usual practice. Acceleromyography using the TOF-Watch® SX [Organon Ireland Ltd., Dublin, Ireland] at the adductor pollicis muscle, monitored neuromuscular block. TOF-Watch® calibration and signal stabilization, using a 5 s 50 Hz tetanic stimulus, occurred after induction of anesthesia, before administration of NMBA or performing any measurements [14]. The device calibrated automatically, providing supramaximal stimulation and optimal gain for each case. We recorded and reported uncorrected (not normalized) TOF ratios, in accordance with consortium guidance [14].

After the last dose of NMBA, patients received study medication intravenously via 2 syringes in masked fashion as a bolus within 20 s. Study medication injection occurred within 2 min of detection of reappearance of T2 for moderate neuromuscular block, or within 2 min of detection of a post-tetanic twitch for deep neuromuscular block. Neuromuscular monitoring continued at least until the subject reached TOF ratio ≥ 0.9, or for at least 30 min following the administration of study drug. If there is a failure to reach TOF ratio ≥ 0.9 within 30 min, the investigators were free to act according to their local clinical practice (e.g., continued monitoring with TOF-Watch SX until TOF ratio ≥ 0.9 was reached, use of an alternative NMTM device, or decision to extubate based on clinical signs).

### Study endpoints

The primary efficacy endpoint, time to recovery of a TOF ratio ≥ 0.9, was compared for ABW vs IBW dosing, pooled across depth of block and NMBA. We also calculated the pooled proportion of patients with prolonged recovery, defined as ≥10 min to recover TOF ratio to  ≥ 0.9.

The main safety outcome compared incidences of treatment-related arrhythmias, including sinus bradycardia and sinus tachycardia, for pooled ABW vs IBW groups. For arrhythmia detection, continuous electrocardiogram monitoring began ≥5 min before study medication administration and lasted ≥30 min thereafter, via caregiver observation and arrhythmia alarms. The proportion of subjects with each of the following treatment-emergent arrhythmias, sustained for ≥1 min after administration of study medication, were compared: sinus bradycardia, defined as a heart rate < 60/min and > 20% less than baseline; sinus tachycardia, defined as a heart rate ≥ 100/min and > 20% more than baseline; and other arrhythmias, defined as a new or worsened arrhythmia, e.g., atrial tachycardia or fibrillation. Other pre-specified safety events were: hypersensitivity, anaphylaxis, and clinically relevant arrhythmias, defined as those necessitating intervention, as determined by the blinded investigator. An external clinical adjudication committee of anesthesia and allergy experts, blinded to treatment, classified potential cases of hypersensitivity and/or anaphylaxis.

### Statistical analysis

Efficacy analyses included all randomized patients dosed with both NMBA and reversal agent who underwent ≥1 post-randomization efficacy assessment. The primary analysis, comparing pooled ABW to pooled IBW groups, employed a stratified log-rank test adjusted for depth of block (moderate or deep) and NMBA (vecuronium or rocuronium). A Cox proportional hazards model, stratified by depth of block and NMBA, estimated the treatment difference between pooled ABW and IBW treatments, reporting a hazard ratio with its 95% confidence interval (95% CI). The median time, with 95% CI, for each group to recover to TOF ratio ≥ 0.9 is reported, based on the Kaplan-Meier method. For the proportion of patients with prolonged recovery the Miettinen and Nurminen method [15] compared the difference between pooled ABW and IBW treatments, reporting 95% CIs of the difference. Patients with a TOF ratio < 0.9 within the observed period were censored from the primary analysis (time to recovery to a TOF ratio ≥ 0.9) but the proportion of patients with prolonged recovery utilized imputed values (See Additional file 2). The primary efficacy hypothesis was tested at 0.05 alpha, 2-sided. Since the primary efficacy hypothesis is related to a comparison of the two dosing paradigms (ABW- vs IBW-based dosing) for a single primary endpoint, there is only one corresponding *p*-value upon which the efficacy conclusion of the study is based. Multiplicity adjustments were not performed due to the single primary hypothesis tested in this study.

Safety analyses included all randomized subjects who received study medication. Comparisons between groups used the stratified Miettinen and Nurminen method; for treatment-related arrhythmias only, we calculated nominal significance levels, without adjustment for multiplicity. The recovery times for each of the pooled ABW and the pooled IBW groups are assumed to follow a normal distribution in the log scale with a median time-to-recovery of 2.2 min [standard deviation (SD): 1.3] for ABW subjects and 3.3 (SD: 2.3) for IBW subjects. Based on the simulated data, using a log-rank test with alpha 0.05, 80 subjects per pooled group (across NMBA and depth of block) provided > 99% power to detect a median difference of ≥1 min in time-to-recovery, i.e., for ABW vs IBW. To populate equally 2 sugammadex ABW groups, 2 sugammadex IBW groups, and 1 neostigmine group, we sought 200 subjects. The number of safety events did not impact sample size calculation, as the safety objectives were to estimate incidences. Between-group comparison nominal *P*-values are provided for reference. Efficacy subgroup analyses by NMBA and depth of block could be underpowered.

## Results

**Fig. 1:**
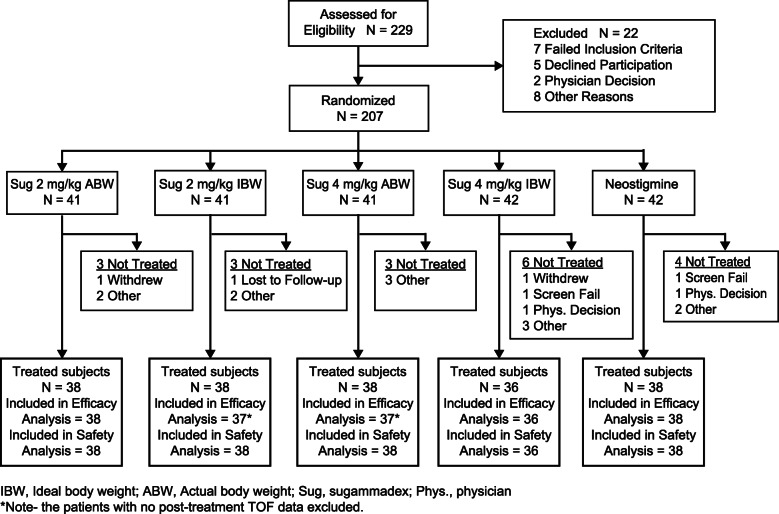
Modified CONSORT diagram showing patient flow during the study

Twenty-one sites in 5 countries screened 229 patients, of whom 207 were randomized at 20 sites (Fig. 1). Of those randomized, 188 received treatment, 186 provided efficacy data, one died post-operatively, and 185 completed all protocol visits. Participants distributed evenly by demographic characteristics across treatment groups (Table 1). Treated patients were 47 years old (median); 11% ≥ 65 years; and 72% women. BMI, ABW, and IBW depicted a morbidly obese population. Sleeve gastrectomy (30%) and gastric bypass (11%) were the most commonly performed procedures. Pre-existing co-morbid conditions displayed adequate balance across groups; overall 51% had hypertension, 29% sleep apnea syndrome, 26% hypothyroidism, 25% gastroesophageal reflux, 20% drug hypersensitivity, and 18% osteoarthritis.

**Table 1:** Demographics

Characteristics	Sugammadex 2 mg/kg ABW *N* = 38	Sugammadex 2 mg/kg IBW *N* = 38	Sugammadex 4 mg/kg ABW *N* = 38	Sugammadex 4 mg/kg IBW *N* = 36	Neostigmine+ Glycopyrrolate *N* = 38	
Age (years)	48 ± 14	48 ± 15	47 ± 11	49 ± 12	48 ± 14	
Female sex	32 (84)	27 (71)	22 (58)	29 (81)	26 (68)	
White race	36 (95)	34 (90)	36 (95)	33 (92)	34 (90)	
NMBA						
Rocuronium	27 (71)	25 (66)	26 (68)	27 (75)	28 (74)	
Vecuronium	11 (29)	13 (34)	12 (32)	9 (25)	10 (26)	
BMI (kg/m^2^)	45.8 ± 4.5	47.2 ± 5.7	45.4 ± 5.0	46.5 ± 5.7	47.3 ± 4.7	
ABW (kg)	127 ± 21	135 ± 17	131 ± 20	131 ± 21	135 ± 20	
IBW (kg)	63 ± 7	65 ± 7	66 ± 7	63 ± 6	65 ± 8	

Subject’s ideal body weight is based on the gender category recorded at the time of randomization

Data entries are either *n* (%) or mean ± SD

*BMI* body mass index, *ABW* actual body weight, *IBW* ideal body weight, *NMBA* neuromuscular blocking agent, *SD* standard deviation

**Fig. 2:**
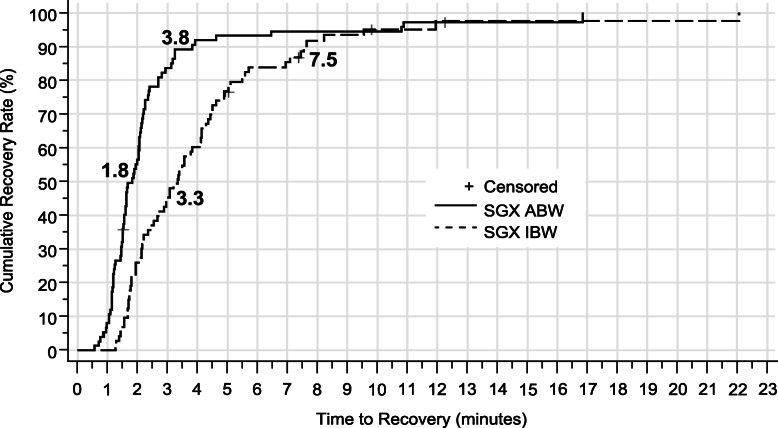
Cumulative percentage of patients achieving TOF ratio ≥ 0.9 pooled across depth of block. The 50th and 90th percentile times, in minutes, are marked for the ABW and IBW pooled groups. A given patient’s results were censored if monitoring terminated or data became unreliable

**Fig. 3:**
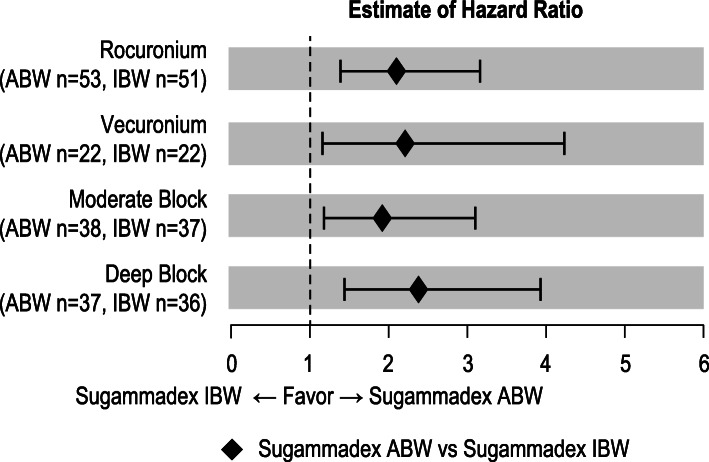
Recovery time to TOF ratio ≥ 0.9 for subgroups. Point estimates and 95% CI presented for respective pooled ABW and IBW groups

Time to recovery to a TOF ratio ≥ 0.9, pooled across NMBA and depth of block, was faster with sugammadex dosed by ABW (median time 1.8 min, 95% CI 1.6 to 2.1) than by IBW (median time 3.3, 95% CI 2.6 to 4.1) (Fig. 2). The resulting comparison was significant (*P* < 0.0001) with hazard ratio 2.13, 95% CI 1.50 to 3.01. Median times to TOF ratio ≥ 0.9 after moderate block were 1.7 min (95% CI 1.5 to 2.1) for ABW, 3.4 min (95% CI 2.2 to 4.4) for IBW, and 34.5 min (95% CI 27.0 to 67.4) for neostigmine. Twenty-four of 186 patients had TOF ratio recorded > 15 min after recovery to 0.9. Re-curarization, i.e., subsequent TOF ratio < 0.9, occurred in 2 of these 24 patients: 1 of 2 patients so monitored in the neostigmine group; 1 of 4 such patients in the 2 mg/kg IBW group; in none of 8 patients in the 4 mg/kg IBW group; and in none of 10 patients in the ABW groups. Neither the choice of NMBA (rocuronium or vecuronium) nor the depth of block (moderate or deep) impacted the effect of ABW vs IBW on the time to reversal (Fig. 3).

Recovery times in the 10% of subjects with the slowest recovery dosed by ABW were ≥ 3.8 min compared with ≥7.5 min for the 10% with slowest recovery dosed by IBW (Fig. 2). Those dosed by ABW took 59% of the time it took those dosed by IBW to achieve recovery. For recoveries to TOF ratios ≥0.8 and ≥ 0.7, the percentages were 60 and 66%. The faster recovery times with ABW vs IBW were consistent for rocuronium and vecuronium. The proportion of patients with prolonged recovery did not differ between ABW and IBW for either moderate block (7.9% ABW; 5.4% IBW; 95% CI of difference [− 10.8, 16.9]) or deep block (2.7% ABW; 0% IBW; 95% CI of difference [− 7.2, 14.2]); however, each moderate block sugammadex group had substantially fewer prolonged recovery patients compared to the neostigmine group (84%).

Table 2 summarizes safety events. Incidences of treatment-emergent bradycardia and other arrhythmias distributed evenly between ABW and IBW groups, pooled across depth of block and NMBA, and across the 5 treatment groups. Although treatment-emergent tachycardia appeared less evenly distributed between ABW (9/76 patients [11.8%]) and IBW (4/74 patients [5.4%]) pooled groups, the difference was not significant (nominal *P* = 0.149). Clinically relevant tachycardia occurred in zero or one patient per treatment group. No significant between-group difference emerged for any treatment-emergent safety endpoint when pooled across depth of block and NMBA, or separately by NMBA.

**Table 2:** Safety results

	Sugammadex 2 mg/kg ABW *N* = 38	Sugammadex 2 mg/kg IBW *N* = 38	Sugammadex 4 mg/kg ABW *N* = 38	Sugammadex 4 mg/kg IBW *N* = 36	Neostigmine + Glycopyrrolate *N* = 38
	*n* (%)	*n* (%)	*n* (%)	*n* (%)	*n* (%)
TREATMENT-EMERGENT EVENTS					
Sinus Bradycardia	2 (5.3)	1 (2.6)	2 (5.3)	2 (5.6)	1 (2.6)
Sinus Tachycardia	4 (10.5)	3 (7.9)	5 (13.2)	1 (2.8)	3 (7.9)
Other Arrhythmias	0	1 (2.6)	0	0	1 (2.6)
EVENTS OF CLINICAL INTEREST					
Adjudicated Hypersensitivity	0	0	0	1 (2.8)	0
Adjudicated Anaphylaxis	0	0	0	0	0
Clinically Relevant ↓ HR	0	0	0	0	0
Clinically Relevant ↑ HR	0	0	0	0	1 (2.6)
Clinically Relevant Arrhythmia	0	1 (2.6)	1 (2.6)	0	0
OTHER ADVERSE EVENTS					
Drug-related Adverse Event	0	2 (5.3)	0	2 (5.6)	6 (15.8)
Serious Adverse Event	1 (2.6)	2 (5.3)	0	3 (8.3)	3 (7.9)
Death	0	0	0	0	1 (2.6)

Pre-defined safety events reported up to 7 days post-dose occurred rarely and evenly across treatment groups: no patient in the sugammadex 2 mg/kg ABW group had an event compared with one (≤2.8%) each in the other groups. For clinically relevant arrhythmias, no subject had bradycardia, one neostigmine patient had tachycardia, and one each in the sugammadex 2 mg/kg IBW and 4 mg/kg ABW groups had other arrhythmias. Of 11 cases of hypersensitivity or anaphylaxis adjudicated, none was anaphylaxis, 2 were hypersensitivity: one of bronchospasm (4 mg/kg IBW) and another rash/pruritis (2 mg/kg IBW).

The numbers/percentages of patients with adverse events (AEs), up to 7 days post-treatment were similar between pooled ABW and IBW groups, and among the 5 treatment groups. No serious AEs were reported and no treated patient, aside from one who died post-operatively, exited the trial prematurely due to an AE. No patient in either ABW group had a drug-related AE; 6 of 10 patients among the other 3 groups with drug-related AEs had been randomized to neostigmine. The post-operative death, from myocardial infarction, assessed by the investigator as not related to study medication, occurred in the neostigmine group.

## Discussion

Comorbid conditions and possible alterations in pharmacokinetics and pharmacodynamics of drugs make the perioperative management of morbidly obese patients difficult. The use of IBW-based dosing of the neuromuscular block reversal agent sugammadex may be considered as a potential cost-saving strategy compared to ABW-based dosing. The present study assesses the safety and efficacy of IBW- and ABW-based dosing of sugammadex for reversal of moderate and deep neuromuscular block in morbidly obese patients.

The current recommended ABW-based dosing of sugammadex for both moderate and deep block derives from studies conducted in patients not morbidly obese [11, 12, 16, 17]. Prior studies of sugammadex reversal of neuromuscular block in the obese studied a mixed population of obese (BMI < 40 kg/m^2^) and morbidly obese (BMI ≥40 kg/m^2^) patients [18–21]. Some trials were observational in design [18, 20, 21]. One trial used ABW to determine the dose for each subject [20]; another used ABW versus a variety of doses greater than IBW [19]; yet another used IBW-based dosing of sugammadex 2 or 4 mg/kg for all subjects followed by 2 mg/kg if recovery had not occurred within 2–3 min [21]. The results of these studies are difficult to compare due to the different study designs and dosing regimens. One prior study concluded that the dose calculated by IBW was insufficient to reverse both moderate and deep neuromuscular blockade [21].

Results of the current study demonstrate IBW-based dosing led to a statistically significant delay in the recovery of neuromuscular function compared with ABW-based dosing in patients with morbid obesity. While the median time to clinical recovery (TOF ratio ≥ 0.9) was 1.5 min faster with ABW vs IBW dosing, pooled across depth of block and choice of NMBA (1.8 vs 3.3 min, respectively), it took ≥3.8 min vs ≥7.5 min for the 10% of patients with the slowest recoveries as dosed by ABW and IBW, respectively. Nevertheless, the clinical value of a few minutes faster reversal may be negligible and will vary among clinicians.

ABW dosing did not result in numerically more frequent treatment-emergent heart rate or rhythm changes, or in more frequent hypersensitivity or anaphylaxis, within the constraints of the sample size assessed. Nor did the overall AE profile of ABW-dosed patients differ from that of IBW-dosed patients, or from the non-morbidly obese patients previously studied [11, 12, 16, 17] . Theoretical concerns of potentially more frequent heart rate, heart rhythm, hypersensitivity, and anaphylaxis occurrences when dosing sugammadex in the morbidly obese by ABW did not materialize in the current study, suggesting previously reported and uncommon treatment-emergent events are not dose-dependent.

Nevertheless, the principle of “do no harm” would direct dosing information on the side of administering the lowest effective dose. For the reversal agent sugammadex, however, under-dosing presents potential safety concerns for patients, such as inadequate reversal, recurrence of block, and actually an increased risk of postoperative pulmonary complications [22]. Recurrence of neuromuscular block occurred in no ABW-dosed patient, but in one patient in the 2 mg/kg IBW group and in one patient of the neostigmine group.

Errors occur frequently in the administration of medications in hospital settings [23, 24] . A dosing recommendation for sugammadex in which doses for morbidly obese subjects is calculated differently from that for the non-morbidly obese invites the potential errors of incorrect application and of miscalculation. It should be entertained only if data suggest superior safety; these data provide no such suggestion.

The design of the study has three limitations: (1) acceleromyography quantitative monitoring of neuromuscular function was used. It was necessary to utilize the TOF Watch device for assessment of efficacy in this study in order to remain consistent with the standardized approach that had been taken throughout the entire sugammadex development program, which included 26 efficacy trials utilizing the TOF Watch [13]. Use of the TOF Watch approach for this study was also an expectation of the FDA. The shortfalls of TOF-Watch technology have been summarized previously [14]. Briefly, acceleromyography can overread the TOF ratios resulting in idiosyncratic values > 1.0, thus overestimating the degree of recovery and underestimating reversal time [25]. Normalization of TOF ratio data can address this issue [26]. Instead of normalization, the protocol required a rigorously-performed calibration. This rigor was successful: the mean normalized TOF ratio was 0.93 ± 0.08 and the investigators rated the native TOF ratio > 0.9 without any differences between the treatment groups. Thus, use of native acceleromyography measurements was unlikely to affect results. (2) IBW and ABW differ by a factor of ~ 2, values at the extremes of various dosing paradigms. The study cannot address whether doses between these two extremes, e.g., a lean body weight-based calculation, might have reversed block as fast as ABW-based doses. The regulatory authority mandated this study to probe safety as well as efficacy, selecting ABW-based dosing to provoke a potential safety signal. No such signal emerged. (3) The protocol did not include re-curarization as an outcome and consequently did not specify a minimum time of neuromuscular monitoring after recovery of the TOF ratio > 0.9. Therefore, it cannot answer definitively if ABW dosing of sugammadex avoids re-curarization.

## Conclusion

In summary, dosing by IBW afforded no incremental safety advantage detectable in this study but did lead to delayed recovery times compared to dosing by ABW. Morbidly obese patients undergoing surgery with neuromuscular block via rocuronium or vecuronium can receive sugammadex dosed as done for other patients, i.e., by ABW, irrespective of the depth of block or choice of NMBA.

## Supplementary Information

**Additional file 1:.** Protocol 146

**Additional file 2:.** Method for Imputation of Missing Recovery Times

**Additional file 3:.** List of Investigators for Protocol 146

## Data Availability

The data sharing policy of Merck Sharp & Dohme Corp., a subsidiary of Merck & Co., Inc., Kenilworth, NJ, USA’s, including restrictions, is available at http://engagezone.msd.com/ds_documentation.php. Requests for access to the clinical study data can be submitted through the EngageZone site or via email to dataaccess@merck.com.

## Abbreviations

ABW: Actual body weight
BMI: Body mass index
FDA: Food & Drug Administration
IBW: Ideal body weight
NMBA: Neuromuscular blocking agents
TOF: Train of four
