# Supplementary material for: Actual versus ideal body weight dosing of sugammadex in morbidly obese patients offers faster reversal of rocuronium- or vecuronium-induced deep or moderate neuromuscular block: a randomized clinical trial
Source: BMC Anesthesiol. 2021 Feb 27;21:62. doi: 10.1186/s12871-021-01278-w (PMC7913453; doi:10.1186/s12871-021-01278-w)
Supplement: Supplementary file 2 — Additional file 2:. Method for Imputation of Missing Recovery Times [file 12871_2021_1278_MOESM2_ESM.pdf]

Additional file 2: Method for Imputation of Missing Recovery Times

If the time from the start of administration of study drug to recovery of the TOF ratio to 0.9 was missing, the imputation was performed as follows for the three potential missing scenarios:

1. Time to TOF ratio to 0.8 was available:

- Sugammadex group: first, for all subjects randomized to receive sugammadex and with times to recovery of the TOF ratio to 0.8 and 0.9 available, the difference between these two recovery times was calculated. Next, the 95th percentile (P95) of these differences was added to the time to recovery of the TOF ratio to 0.8 of the subjects with missing times to recovery of the TOF ratio to 0.9. This was used as the imputed missing time to recovery of the TOF ratio to 0.9.

- Neostigmine group: same as for the sugammadex group but now only subjects randomized to receive neostigmine were used, and the 5th percentile (P5) of the differences in time to recovery of the TOF ratio to 0.8 and 0.9 was calculated.

2. Time to TOF ratio to 0.7 is available, but the time to TOF ratio to 0.8 is missing:

- Sugammadex group: first for all subjects randomized to sugammadex and with times to recovery of the TOF ratio to 0.7 and 0.9 available, the difference in time between these two recovery times was calculated. Next, the P95 of these differences was added to the time to recovery of the TOF ratio to 0.7. This was used as imputation of the missing time to recovery of the TOF ratio to 0.9.

- Neostigmine group: same as for sugammadex group but now only subjects randomized to receive neostigmine were used and the P5 of the differences in time to recovery of the TOF ratio to 0.7 and 0.9 was calculated.

3. Times to TOF ratio to 0.7 and to 0.8 are both missing:

- Sugammadex group: the P95 of the time to recovery in all subjects randomized to sugammadex with an observed recovery time of the TOF ratio to 0.9 was imputed.

- Neostigmine group: the P5 of the time to recovery in all subjects randomized to neostigmine with an observed recovery time of the TOF ratio to 0.9 was imputed.

The imputation was performed across NMBA and depth of blocks, and by ABW and IBW for the sugammadex group. The imputation of missing time generated from the above procedures were calibrated further with the time of last reliable TOF ratio time as taking the maximum of: 1) the imputed time from the above procedures, and 2) the time to the last reliable ratio.
