# Supplementary material for: Actual versus ideal body weight dosing of sugammadex in morbidly obese patients offers faster reversal of rocuronium- or vecuronium-induced deep or moderate neuromuscular block: a randomized clinical trial
Source: BMC Anesthesiol. 2021 Feb 27;21:62. doi: 10.1186/s12871-021-01278-w (PMC7913453; doi:10.1186/s12871-021-01278-w)
Supplement: Supplementary file 3 — Additional file 3:. List of Investigators for Protocol 146 [file 12871_2021_1278_MOESM3_ESM.pdf]

Additional File 3: List of Investigators for Protocol 146

Country	Independent Ethics Committee	Principal Investigator
Austria	Ethik-Kommission Der Stadt Wien	Walter Klimscha (Sozialmedizinisches Zentrum Ost – Donauspital, Wien, Austria)
Belgium	Universitair Ziekenhuis Antwerpen Ethisch Comite	Vera Saldien (Universitaire Ziekenhuis Antwerpen – UZA, Edegem, Belgium)
Denmark	De Videnskabsetiske Komiteer For Region Hovedstaden	Christian Sahlholt Meyhoff (Bispebjerg og Frederiksberg Hospital, Copenhagen, Denmark)<br><br>Torsten Lauritsen (Rigshospitalet, Copenhagen, Denmark)
Germany	Ethik-Kommission der Landesaerztekammer Baden-Wuerttemberg	Manfred Blobner (Klinikum Rechts der Isar Technische Universitaet Muenchen, Munchen, Germany)<br><br>Alexander Reich (Josephs-Hospitals Warendorf, Warendorf, Germany)<br><br>Friedrich Puehringer (Klinikum am Steinenberg Reutlingen, Reutlingen, Germany)<br><br>Georg Baumgarten (Johanniter Krankenhaus Bonn, Bonn, Germany)<br><br>Michael Moellmann (St. Franziskus-Hospital, Muenster, Germany)<br><br>Michael Przemeck, PD

		(Diakovere Annastift gGmbH, Hannover, Germany)<br><br>Hinnerk Wulf (Universitatsklinikum Giessen und Marburg GmbH, Marburg, Germany)<br><br>Niels Rahe-Meyer, PD (Franziskus Hospital Bielefeld, Bielefeld, Germany)
United States	University of California Davis Medical Center IRB	Richard L. Applegate, M.D. (University California / Davis, Sacramento, CA)
	Western Institutional Review Board	Neil W. Brister (Temple University Hospital, Philadelphia, PA)<br><br>Keith Candiotti, M.D. (Jackson Memorial Hospital/University of Miami, Miami, FL)<br><br>Roy G. Soto (William Beaumont Hospital - Royal Oak, Royal Oak, MI)<br><br>James Tse (Robert Wood Johnson University Hospital, New Brunswick, NJ)
	Zablocki VA Medical Center IRB	Thomas Jay Ebert (Zablocki VA Medical Center, Milwaukee, WI)
	Mission Health IRB	Shannon Elizabeth Meron (Mission Hospital – St. Joseph, Asheville, NC)
	Copernicus Group Independent Review Board	David Leiman (Hermann Drive Surgical Center, Houston, TX)
	Cleveland Clinic Institutional Review Board	Kurt Ruetzler, M.D. (Cleveland Clinic Foundation, Cleveland, OH)
	Vanderbilt Human Research Protection Program	Edward Read Sherwood

		(Vanderbilt University Medical Center, Nashville, TN)
	University of Kansas Medical Center IRB	Matthew Wyatt (University of Kansas Medical Center, Kansas City, KS)
	Copernicus Group Independent Review Board	Daneshvari R. Solanki (Hermann Drive Surgical Center, Houston, TX)
	MU Institutional Review Board	Boris Mraovic (University Hospital-Columbia MO, Columbia, MO)
